# An unusual case of *Candida parapsilosis* endocarditis of the native tricuspid valve secondary to a tunneled dialysis catheter

**DOI:** 10.47487/apcyccv.v4i3.307

**Published:** 2023-09-30

**Authors:** Nathalie Victoria Zacarías Mendoza, Andrea Verónica Zevallos Goyzueta, Alexander Wu Chen, Víctor Justo Robles Velarde

**Affiliations:** 1 Facultad de Medicina Alberto Hurtado, Universidad Peruana Cayetano Heredia. Lima, Peru. Universidad Peruana Cayetano Heredia Facultad de Medicina Alberto Hurtado Universidad Peruana Cayetano Heredia Lima Peru; 2 Servicio de Cirugía Cardiovascular, Instituto Nacional Cardiovascular. Lima, Peru. Servicio de Cirugía Cardiovascular Instituto Nacional Cardiovascular Lima Peru

**Keywords:** Endocarditis, Candida parapsilosis, Catheter-Related Infections, Endocarditis, Candida parapsilosis, Infecciones Relacionadas con Catéteres

## Abstract

*Candida* endocarditis is a severe disease associated with high mortality rates. *Candida parapsilosis* is frequently identified as the causative pathogen in intravenous drug users and is commonly associated with nosocomial infections, primarily due to its ability to form biofilms on catheters or other foreign bodies. Here, we present a rare case of *Candida parapsilosis* endocarditis affecting the native tricuspid valve in a 35-year-old male patient with end-stage chronic kidney disease (Stage V), who had a suspected fungal infection related to the left cervical catheter. The patient received treatment with caspofungin and underwent excision of a verrucous tumor on the tricuspid valve. Despite encountering postoperative complications, the patient was discharged on fluconazole treatment and scheduled for follow-up. *Candida* endocarditis poses a clinical challenge that necessitates a multidisciplinary approach and tailored management due to its infrequent occurrence and higher mortality rate compared to bacterial endocarditis.

## RESUMEN

La endocarditis por *Candida* es una enfermedad grave asociada con tasas de mortalidad elevadas. *Candida parapsilosis* se identifica con frecuencia como un patógeno que afecta usuarios de drogas intravenosas y está comúnmente relacionada con infecciones nosocomiales, principalmente debido a su capacidad para formar biopelículas en catéteres u otros cuerpos extraños. Se presenta un caso inusual de endocarditis por *Candida parapsilosis* que afecta la válvula tricúspide nativa en un paciente masculino de 35 años con enfermedad renal crónica en etapa terminal (Etapa V), quien tenía una sospecha de infección fúngica relacionada con el catéter cervical izquierdo. El paciente recibió tratamiento con caspofungina y se sometió a la extirpación de un tumor verrugoso en la válvula tricúspide. A pesar de enfrentar complicaciones posoperatorias, el paciente fue dado de alta con tratamiento de fluconazol y se programó un seguimiento. La endocarditis por *Candida* presenta un desafío clínico que requiere un enfoque multidisciplinario y un manejo personalizado debido a su ocurrencia infrecuente y una tasa de mortalidad más alta en comparación con la endocarditis bacteriana.

## Introduction

*Candida* infectious endocarditis, although accounting for less than 5% of all cases, is a severe disease characterized by increasing incidence and high mortality rates. Among the non-albicans species of *Candida, Candida parapsilosis* is the most commonly associated species with this condition. Patients with chronic kidney failure and long-term catheters are considered at high risk ^1^. We report a rare case of *Candida parapsilosis* endocarditis affecting the native tricuspid valve in a 35-year-old male patient with end-stage chronic kidney disease (Stage V) who has been undergoing hemodialysis with a probable fungal infection of the left cervical catheter.

## Case report

**Figure 1:**
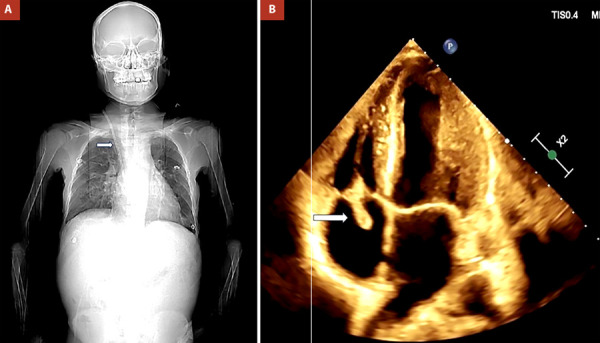
A. Coronal view of computed tomography shows tunneled central venous catheter (arrow). B. Transthoracic echocardiogram, demonstrating a pedunculated mass (20 x 16 mm) on the postero-lateral leaflet of the tricuspid valve (arrow).

A 35-year-old male patient was admitted to our Emergency Department with a diagnosis of *Candida parapsilosis* endocarditis. Regarding his medical history, the patient had end-stage chronic kidney disease (Stage V) and had been on hemodialysis since the age of 25, with multiple changes of accesses and catheters since 2012 (cervical, right femoral, left femoral, Tenckhoff, tunneled central venous catheter (CVC), and temporary catheters). He also had a history of hypertension treated with amlodipine 10 mg since the age of 25, an appendectomy at the age of 18, and a gastric ulcer. Three months before admission, the patient was hospitalized due to hypovolemic shock, and the left cervical catheter was removed due to a probable fungal infection **(**Figure 1A**)**. One month before admission, the patient presented to his local hospital with a persistent febrile condition, pancytopenia, and acute gastroenterocolitis. Blood cultures were positive for *Candida parapsilosis* sensitive to caspofungin. Transthoracic echocardiogram (TTE) **(**Figure 1B**)** revealed suggestive images of infectious endocarditis, including a pedunculated mass measuring 20 x 16 mm on the postero-lateral leaflet of the tricuspid valve, attached to the atrial surface, causing moderate insufficiency, and circumferential laminar pericardial effusion.

The patient was admitted to the emergency department with the following diagnoses: infective endocarditis of the tricuspid valve caused by *Candida parapsilosis*, moderate tricuspid regurgitation, moderate to severe pancytopenia, and CKD (Stage V) on hemodialysis/anuria due to the underlying condition.

Upon admission, the patient presented with a subfebrile condition that had been evolving for 2 months, with an insidious onset and progressive course. He was hemodynamically stable, receiving oxygen support (4 L/min nasal cannula), and undergoing treatment with caspofungin 50 mg/24 hours IV (on the 6th day), gentamicin 150 mg/48 hours IV (on the 7th day), vancomycin 1 g/72 hours IV, and post-dialysis vancomycin 1 g (on the 11th day).

Physical examination on admission revealed a male patient with an ectomorphic build (height: 145 cm; weight: 41 kg; body mass index: 19.5 kg/m^2^). Blood pressure was 144/78 mm Hg, heart rate 81 bpm, respiratory rate 12 rpm, oxygen saturation 96%, and temperature 37 °C. The patient was in poor general condition, breathing spontaneously, with a right jugular CVC, hematomas in the right and left inguinal areas, grade III/VI systolic murmur at the tricuspid area, jugular engorgement, bilateral crackles predominantly at the bases of 2/3 of the lung fields, tense globular abdomen with collateral circulation, positive fluid wave, and hepatomegaly.

Initial arterial blood gas analysis showed pH 7.38, pO_2_ 72 mmHg, FiO_2_ 0.28, and pCO_2_ 42 mmHg. The electrolyte panel and complete blood count revealed potassium (K) 5.5, sodium (Na) 134, lactate 0.9, and hemoglobin (Hb) 7.5. TTE showed an ejection fraction (EF) of 69%, a left atrial volume index (LAVI) of 61, and an isoechoic image measuring 22 x 23 mm on the posterior leaflet, fixed on the atrial side, causing moderate to severe regurgitation.

During the ICU stay, antibiotics were discontinued. The patient presented pancytopenia without signs of bleeding, so 6 platelet apheresis transfusions were administered, and intermittent hemodialysis was continued. Due to a hemoglobin level of 7.3, a packed red blood cell transfusion was performed. Despite caspofungin coverage, on the 9th day, the patient remained febrile with mild pulmonary congestion and oxygen requirement.

Emergency cardiac surgery was decided. Intraoperatively, transesophageal echocardiography (TEE) also revealed a 25 x 18 mm mass on the posterior leaflet of the tricuspid valve, sessile but with a hypermobile appendix. The surgical approach was performed through a median sternotomy with central arterial and bicaval venous cannulation. During right atriotomy, a 30 x 25 mm tumor was observed on the anterior and posterior leaflets of the tricuspid valve. No masses were found in other locations, and mild to moderate tricuspid regurgitation was observed. Excision of the tricuspid valve and cleaning of the tricuspid leaflet base with iodine solution were performed.

Postoperative TEE confirmed a tricuspid valve with mild thickening of the posterior leaflet, no residual mass, and mild central regurgitation. No other significant valvular pathology was observed, and biventricular contractility was normal. The postoperative period had complications, such as bleeding at the posterior left ventricle and subfebrile condition. The laceration in the posterior wall of the left ventricle was successfully addressed through a conventional resternotomy procedure, using nonabsorbable thread stitches (Prolene®).

On the 4th day, the patient became hemodynamically unstable, requiring dual vasopressor support due to refractory septic shock. Additionally, acute respiratory failure due to ventilator-associated pneumonia developed. A radiological examination revealed an infiltrate in the upper lobe of the right lung, and subsequent culture testing confirmed the presence of *E. coli* with extended-spectrum beta-lactamase (ESBL) resistance, piperacillin-tazobactam was administered.

The patient was discharged on the 48th day postoperatively, exhibiting stable hemodynamics, spontaneous ventilation, a lack of fever, and no reported complications. He was prescribed a 21-day course of fluconazole and scheduled for regular outpatient hemodialysis follow-up appointments.

## Discussion

Infective endocarditis (IE) is commonly caused by bacterial infections, primarily *Staphylococcus*, *Streptococcus*, and Enterococcus ^2^. However, fungal microorganisms such as *Candida* spp*.* and *Aspergillus* spp*.* can also lead to IE ^3^. *Candida* endocarditis (CE) is a rare (1-2%) but life-threatening condition associated with a higher mortality rate compared to bacterial IE ^4^^-^^6^.

CE is typically linked to bloodstream infections by *Candida* or alterations in intestinal flora ^6^. The most common species causing CE are *C. albicans* and *C. parapsilosis*^5^. *C. parapsilosis* has been increasing in prevalence, particularly in intravenous drug users and as a nosocomial infection due to its ability to form biofilms on catheters or foreign bodies ^3^^,^^4^. Predisposing risk factors for *C. parapsilosis* include prosthetic heart valves or devices (57.4%), intravenous drug use (20%), and less frequent factors such as parenteral nutrition (6.9%), immunosuppression (6.4%), broad-spectrum antibiotic treatment (5.6%), and pre-existing valvular disease (4.8%) ^4^. 

In our case, the patient had stage V chronic kidney disease (CKD) and a history of prolonged use of central venous catheters (CVC) for hemodialysis, which are significant risk factors for developing CE ^(^^5^^,^^7^. Interestingly, the infection affected the native tricuspid valve, which is rare considering the strong association of *C. parapsilosis* with prosthetic valves or devices.

The patient presented with persistent fever and symptoms associated with heart failure and pericardial effusion. Unlike bacterial endocarditis, fungal endocarditis is highly associated with embolic and hemorrhagic events (approximately 40-45%) due to the formation of larger vegetations that mainly affect the cerebral area and lower extremities ^4^^,^^8^^-^^10^. Fortunately, the patient did not exhibit these complications and was hemodynamically stable upon admission.

Microbiological testing confirmed *C. parapsilosis* as the causative pathogen, with susceptibility to caspofungin. The latest guidelines from the Infectious Diseases Society of America (IDSA, 2016) and the American Heart Association (AHA, 2015) ^11^^,^^12^ recommend initial treatment for native valve endocarditis as amphotericin B with or without flucytosine or a high-dose echinocandin like caspofungin, along with valve replacement ^7^. In our case, caspofungin was chosen and administered at a dosage of 50 mg/24h IV. However, despite antifungal treatment, the patient remained febrile on the 9th day, possibly due to multiple comorbidities. Therefore, surgical excision of the verrucous tumor on the tricuspid valve was performed.The postoperative period had its challenges, including bleeding at the posterior left ventricle, subfebrile condition, refractory septic shock, and ventilator-associated pneumonia leading to acute respiratory failure. These complications may be associated with the patient’s immunosuppressed state caused by pancytopenia. 

*Candida* endocarditis poses clinical challenges and requires individualized management due to its rarity and higher mortality rate compared to bacterial endocarditis. In our case, the patient presented with an uncommon tricuspid valve infection caused by *C. parapsilosis.* Additionally, several risk factors and comorbidities, such as advanced-stage CKD and prolonged use of catheters for hemodialysis, predisposed the patient to the development of endocarditis, complicating its management. The combination of surgical intervention and antifungal therapy was necessary for the patient’s clinical improvement. According to the “2023 ESC Guidelines for the management of endocarditis: Developed by the task force on the management of endocarditis of the European Society of Cardiology (ESC) Endorsed by the European Association for Cardio-Thoracic Surgery (EACTS) and the European Association of Nuclear Medicine (EANM)”, there are three primary criteria for contemplating surgical intervention in cases of acute infective endocarditis (IE): heart failure, uncontrolled infection, and as a preventive measure to mitigate the risk of septic embolization. In this particular case, the urgency of surgery stemmed from an unmanageable infection caused by a highly virulent pathogen, *C. parapsilosis*^13^*.*

This case emphasizes the importance of considering *Candida* endocarditis as a potential diagnosis, even in atypical cases, and underscores the need for a multidisciplinary approach to managing this disease. Sharing clinical cases like this contributes to medical knowledge and aids in improving outcomes in the treatment of *Candida* endocarditis.
